# Simulating worm feeding patterns with computational models

**DOI:** 10.1038/s41598-024-61165-5

**Published:** 2024-05-09

**Authors:** Neil Vaughan

**Affiliations:** 1 University of Exeter, RILD Building, Barrack Road, Exeter, EX2 5DW UK; 2 Royal Academy of Engineering, London, UK

**Keywords:** Computational models, Computational science

## Abstract

Worms create complex paths when moving through sediment to feed. This research applies computer simulation models to provide a unique approach to visualise and quantify the process by which complex worm paths can emerge from simple local movement decisions. A grid environment is proposed in which worms can move with choice of up to 8 directions at each step. This uses a square grid with diagonal paths which has not been investigated before and the resulting number of complex paths is increased compared to triangular grids. Results identify many novel worm paths. Some of the resulting paths are symmetrical, others produce repetitive looping paths, others return to the origin. Interesting worm paths are identified with chaotic movement. Some include oscillating between chaotic and ordered movement for which the outcome is still unknown after millions of steps. A conclusion that may be extrapolated to other creatures is that local movement decisions of a species substantially determine the overall global search strategy that emerges.

## Introduction

**Figure 1:**
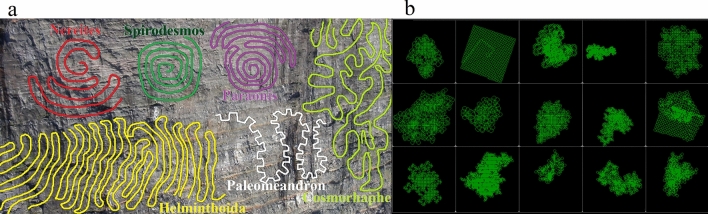
Paths traced by (**a**) various species of worm invertebrate in fossils, (**b**) simulated worms developed in this research.

Bottom feeding ocean invertebrates and worms produce an intricate variety of meandering paths whilst searching for nutrients in sediment. Commonly these paths are difficult for us to observe on the deep ocean floor or underneath silt and sediment layers. However, fossilised remains of worms up to 1200 million years old^1^ provide a clear window for these benthonic invertebrate sediment feeding patterns to be observed^2^. This enables comparisons to be made between feeding patterns^3^ produced by various extinct or living species and genera that burrowed them (Fig. 1a), providing evidence about their behavioural patterns^4^.

Worm feeding paths cover as much ground as possible, without retracing paths that have been previously traversed, as those contain fewer nutrients. Different worm decision rules result in different complex paths being traversed. Simple rules can result in surprisingly complex and intricate paths. However, the complexity of worm paths makes it difficult to determine how those worm paths end and whether the paths return to the place where they started.

Patterns produced by real worms (Fig. 1a) can be very different from each other. Therefore, this research doesn’t aim to exactly replicate these specific 6 example fossil patterns. Instead the aim is to create a simulation model solely using local movement decisions, capable of producing a similar variation of wide ranging, diverse global search behaviour patterns. The worms simulated in this research produce a similar variety of diverse interesting search patterns, as demonstrated by examples shown in Fig. 1b.

### Previous worm computer simulation

A model known as Paterson's worms was introduced using a hexagonal grid in 1971 by Mike Paterson and John Horton Conway^5^, to model the feeding patterns of certain worms. Conventional models using a hexagonal grid^6^ have allowed the worm to choose between 4 or 6 angles when changing direction^7^, which resulted in 412 unique worms^8^. Some of the most complex paths were not solved until a new algorithm based on hashlife^9^ which gave ability to speed up simulations. Currently, more than fifty years later, with substantial analysis, the outcome of one worm still remains unknown: worm code 1042015^10^. This worm is known to be active beyond at least 5.2 × 10^19^ timesteps, which is more than the number of grains of sand on earth. These computer models simulate interesting feeding patterns produced by invertebrates. Since the original publication by Gardner^5^, in over 50 years, there has not yet been any journal or conference publication to follow up on Paterson’s worms or any variation or extension to the original 1973 model.

Prescott and Ibbotson^11^ used physical line following robots to create invertebrate style meandering patterns, and concluded that the intelligent behaviour capacity shown in the patterns of behaviour-based robots resembles that of animals in late pre-Cambrian period.

Various nature-inspired properties can be modelled by computer simulations, such as avoiding paths that have been already traversed, turning at regular intervals, at various angles and keeping close to previously traversed paths which is beneficial to keep the meandering path in a small confined space^2^. By adjusting, tuning or evolving the parameters of simulation, an effect resembling genetic variation is produced, which then controls aspects of the algorithmic behaviour. These parameters can affect properties such as the angle to turn when encountering a path that is already traversed, the distance to keep between a new path and an existing path, and the time interval between turns.

### Contributions of this research

Some main contributions from this work are: (1) The introduction of a new grid type for simulating worms. (2) Several example images are shown of worms on this square grid, which have not been modelled before. (3) A category system to organise worms according to their termination or outcome letter: T, L, R, I, N. (4) A new Eq. (1) is proposed which estimates the maximum number of possible worms on this grid is over 10^33^. The time required to draw all worms that are possible on this new grid at a speed of 10 worms per second (wps), would be longer than twenty trillion times the age of the universe. (5) Highlighted multiple worms of unknown destiny, which appear to have potential to run indefinitely or for an unknown time. (6) All of the 4.5 million worms with rule lengths up to 11 have been run and analysed. (7) A large number (over 33,300) of random worm codes were generated and analysed up to population of 2 million. (8) A proof is developed that no worm can have over 60 rules and in practice no worm has been seen to use over 34 rules.

These simulations provide one of the most clear ways to visualise and quantify how local movement decisions lead to emergence of complex structured global search patterns. This contributes to explaining variations seen in fossil records (Fig. 1a) between the global search strategies of different species. This research also develops methods for categorising the emergent search behaviours into distinct categories by quantifying their properties.

## Methods

### Methods of the novel proposed 8 path grid

The proposed simulation acts within a grid of points on an x, y plane, and has been developed with higher degrees of freedom than conventional hexagonal grid models. This research has developed a new computer simulation environment which allows diagonal turns, enabling greater flexibility, with 8 directions for the worms to turn onto after each step. In practice this is restricted to 7 because path 4 is always unavailable, due to the previous move.

**Figure 2:**
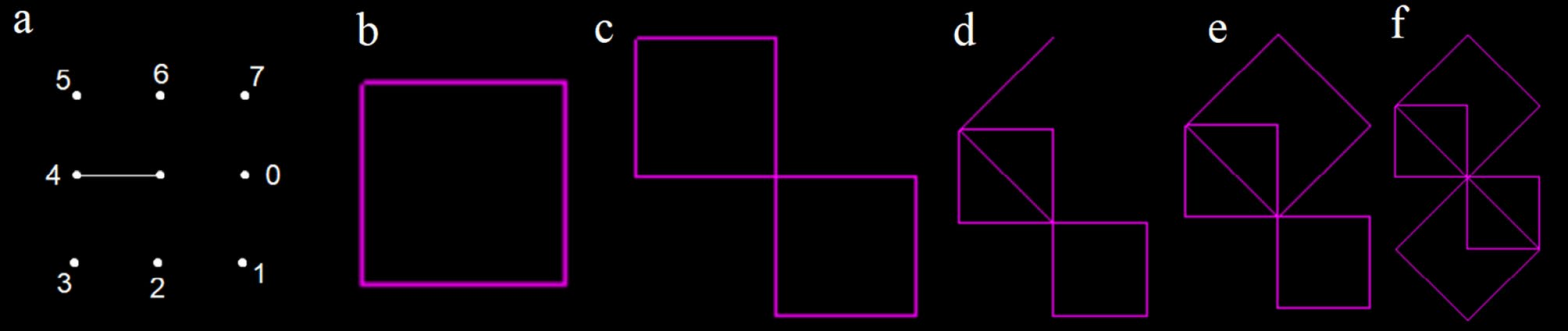
(**a**) Description of numbering of the proposed grid. (**b**–**f**) Five stages detailing the growth process of a path produced by a simple example worm (26326) with five rules.

Simulated movement of worms starts at a central point on an infinite grid. The worm initiates the path by taking one step and arriving at a new point. Thereafter, at each new grid point, eight lines meet. The worm then chooses one of the gridlines to move along. To simulate the worm’s senses, the worm detects which, if any, of the 8 gridlines have already been traversed. The worm can never travel along a line between two points that has already been traversed. Therefore, this produces a choice of up to 7 directions per step (0–3 and 5–7), excluding 4 which is the line traversed on the previous step (Fig. 2a). The worm chooses a path from the available paths. The worm stores it’s decisions in memory. Each time an identical distribution of available paths is encountered again, it must make the same decision. If only one path is available, that path gets taken automatically without any rules. If the worm gets to a dead-end point where all lines have been taken, the worm terminates^5^. The number of lines drawn at the time of termination is referred to as the worm’s population size. Each worm can be described by a genetic code such as 26,326 which refers to a worm with 5 rules and the numbers represent the path numbers taken when each rule is set, as with Kahrkling^12^ notation.

An example of a basic worm in this proposed 8 path model (code 26326) is shown in Fig. 2b–f. The paths are numbered from 0 meaning continue straight forwards, through to 7 meaning 45 degree turn to left (Fig. 2a). All worms are initialised with one line towards the positive x axis. The worm’s first decision rule is to take path 2, so the worm takes a 90 degree turn to it’s right. Number 2 is stored in memory as the first rule. It encounters the same situation twice more, so each time it repeats this previous decision, causing the worm to arrive back at the origin (0, 0), having drawn a square (Fig. 2b). The worm has not encountered this situation before because path 2 is traversed, so this time the worm chooses a new rule, path 6 which is a 90 degree turn to it’s left. The worm then turns right 3 times due to the first rule 2, again arriving at the origin having produced a double square pattern (Fig. 2c). It then chooses a new rule, path 3 which is a sharp 135 degree turn to the right (Fig. 2d). The worm then follows the first rule path 2 three times arriving at the origin for a third time (Fig. 2e). The worm finally chooses a new rule, path 6 (which is one of only two paths remaining) before following the initial rule three times. The worm then terminates because it arrives back at the origin for the fourth time (Fig. 2f) with no more paths are available. Measuring each worm’s population and the number of rules, allows comparison between worms. The worm 26326 in Fig. 2b–f terminates with population 14 and 5 rules.

### Worm categories and worm codes

Five categories for worm termination are proposed. When a worm ends, it can be classed into one of the five categories below, representing the reason why the worm ended:

- T = Terminated because no paths were available by arriving back at the origin (0,0). All child worms of this worm will also be in category T.
- L = A repeating infinite loop was detected, could be of various lengths. All children of this will be the same category.
- R = Running continuously until the population limit of the simulation (perhaps 2 million steps). These worms and all of their child worms could in future be re-run with higher computing power or techniques such as hashlife^9^ to determine their outcomes.
- I = Invalid rule, a rule specified to turn onto a path that is already traversed. All child worms of this will also be category I. These invalid rules are commonly encountered when setting rules in advance, but can be avoided if rules are chosen dynamically at runtime, or during recursive search if the parent worm has already been completed with code N.
- N = Need more rules. The worm has used all of the specified rules but another rule was required. All children of this need to be run with additional rules to determine their outcomes.

Five different methods were implemented for generating unique worm rules. (1) Inputting a pre-set rule, where a complete worm rule, as a string such as 26326, is entered in advance and run. Some of these rules may be invalid. (2) Brute force by running every worm including invalid worms, by generating all worm rules from 0 to 7 at every level. The resulting number of worms is found by the equation $\sum_{n=0}^{i-1}3({7}^{n})$, where *i* is the rule length limit. This shows that there would be 342,947 worms with maximum rule length of 7 generated by this method. A substantial proportion (over 90%) of worms generated using brute force method will be category I invalid, as it is known that there are 28,305 valid worms up to rule length 7 (Table 1). (3) Randomly generating the next part of the rule when it’s required at run-time by randomly choosing an available path. This avoids all category L and N worms and can run to any rule depth, ending in three categories: T, L or R. (4) Recursive with pruning, which explores only available paths and only generates children within worms of category N. This can be run with a rule length limit such as all worms with rules to maximum length of 11. (5) Interactive evolutionary computation (IEC) where a message prompt allows the user to select a rule from available paths at run-time, which is available on the accompanying website^13^ (Fig. 9).

**Table 1:** Results from recursive run of all worms up to length of 11 rules with population limit of 250,000.

Worm category	Number of rules										
	1	2	3	4	5	6	7	8	9	10	11
N	3	21	104	457	1816	7143	27,221	101,175	367,557	1,296,123	4,420,602
T	0	0	0	0	15	56	240	1104	4273	16,218	59,674
L	0	0	2	18	71	220	843	2595	7249	20,631	59,793
R	0	0	0	0	0	0	1	3	43	146	436
Total	3	21	106	475	1902	7419	28,305	104,877	379,122	1,333,118	4,540,505

This table shows the number of worms in each category (N, T, L, R) in the left row, for worms of each rule length (1–11 shown in the top row).

### Estimating the total number of unique worms

There are mirror image worms which can be excluded as duplicates. The first rule is restricted to paths 1, 2 or 3, when generating random or recursive worm codes. This avoids paths starting with 0 which only draws a straight line and path 4 which is already traversed. Also this avoids paths 7, 6 and 5 which are exact mirror images^7^ of 1, 2 and 3 respectively. Every worm code can be converted into a mirror image by converting the worm code, such that every rule digit 1, 2 or 3 becomes a 7, 6 or 5 respectively.

A proof has been developed to show that any worm on this grid will always be limited to use no more than 60 rules. The first rule is restricted to 1, 2 or 3, to avoid mirror images. The 2^nd^ rule always occurs at the origin with 6 paths available. All other rules including the third rule onwards, have 5 or less paths available. Worms have 7 sensors which have two states (binary): 1 or 0. The maximum number of rules is therefore 2^7^ = 128 rules. For each of the 128 binary 7 digit path distributions, the number of paths available is the Hamming weight of the binary string (inverted so that a 1 represents an available path and a 0 represents a traversed path). Half of those, 64 apply to situations with an even number of paths available, for example 00001100, (which becomes 1111011 when inverted and ignoring path 4) and all others where the Hamming weight of the inverted binary string is even. In practice, the number of available paths is always odd, except when the worm is exactly located at (0, 0), which can only occur and result in a new rule a maximum of three times per worm. Of the 64 odd rules, 1 has 7 paths, 21 have 5 paths, 35 have 3 paths and 7 have 1 path. The 1 rule with 7 paths is 0000000, the initial rule which is restricted to 3 (paths 1, 2 or 3). The 7 situations with 1 path are automatically selected without a rule. This leaves a maximum of 57 odd rules. Additionally a worm could potentially also use a maximum of 3 rules with an even number of paths and on those three occasions the number of available paths are known to be 6, 4 and 2 respectively. Therefore it is not possible for any worm on this grid to use more than 60 rules.

Estimating the total number of unique worms on this grid helps to reveal the extent of variety, the full number of rulesets, or genetic pool. In order to estimate this, there are certain facts that restrict the total number. The fact that one worm cannot use more than 60 rules restricts the total number of worms. The number of available paths in each rule is used in the Eq. (1).

1 $$3*6*{5}^{21}*{3}^{35}*4*2 = 48*{5}^{21}*{3}^{36} = 3.43*{10}^{33}$$

Equation (1) gives the maximum possible number of unique worms of length 60. If all parent worms of length 1–59 are also included this increases to over 6*10^33^. In practice no rule length above 34 has been identified, so if rule length is limited to 34, this would be up to 1.5*10^20^ unique worms of length 34.

Estimating the number of worms including parents up to a given length is also useful. If all worms are run to a limit of rule length such as 7, it is useful to estimate how many valid worms there will be. The order that the number of paths per rule will occur in for a particular worm is unknown, but we can assume that all rules from the third rule onwards have 5 paths. Therefore the number of unique worms with length *i* = 7 is proven to be not more than 3*6*5*5*5*5*5 = 3*6*5^i-2^ = 18*5^5^ = 56,250. Furthermore if all parent worms are included, this becomes 3 + (3*6) + (3*6*5) + (3*6*5*5) + …. + (3*6*5^i-2^) = 70,311 unique worms of lengths 1 to 7.

### Method for automatic detection of looping patterns

A method was developed to detect looping patterns during simulation. The benefits are: to enable early halting of a long running looping worm, avoid needing to run the children of looping worms and to avoid having to manually identify and label looping worm patterns. This works by searching within the stored sequence of rule activations. Patterns must repeat at least 4 times to be detected. Therefore the longest loop that can be detected is of length up to ¼ population size limit. This detects repeating loops consisting of any length including small or large complex sections with minimum length of 6 characters to avoid false detection of temporarily looping short sections. The method also enables counting the lengths of each loop, to identify which worms have the most complex repeating loop consisting of a large repeating section. The algorithm takes a substantial time to run when worms are of a long length, so the loop detection algorithm is only run once every few hundred thousand steps to reducing computation time.

Worms will always have a path back to the origin, so can never become blocked off from the origin centre point. Whenever a worm takes 1 step into a layer further away from the origin, the step’s even pair will be available to return to the origin. The number of paths outward from the origin in every layer is therefore even, given by the formula $24{\text{n}}-16$ where n is the layer number or the shortest distance to origin.

## Results

### Software development

Source code was developed in various languages. A JavaScript version of the worms simulator was developed which can be run in any web browser. This allows worms to be simulated by any user with access to a web browser on any platform, including Apple, Samsung, smartphones, iPhones, Windows, PCs, Linux or other platforms. An ANSI C version of the code was developed and compiled into a windows 64-bit .exe file which can simulate the worms at faster speeds and higher population sizes up to 10 million. A website was developed^13^ to host the JavaScript worms implementation along with several example images of complete worms of various sizes and categories. The website also has an Interactive Evolutionary Computation (IEC) version (Fig. 9) with instructions for use and result tables containing the worm categories and image files in .PNG format for all 1902 worms with rule lengths between 1 and 5 (Table 1).

### Minimum number of rules and population size

All worms with 1 or 2 rules are in category R. The shortest worms in category L infinite loops have rule length 3, which are worms 337 and 377. The shortest worms in category T have rule length 5, such as worm 26326 in Fig. 2b–f. For worms that terminate T, the smallest possible population size is 14 (Table 4). Every population size from 14 to at least 225 has been seen to occur in category T.

### Verifying the maximum number of rules used by one worm

Currently no worms have been identified yet that use more than 34 rules. This appears to be because worms infrequently encounter 34 different scenarios before terminating, or hitting an invalid rule when using pre-set rules, or beginning a loop. This fits with the proof that no worm can use more than 60 rules. If a larger number of random worms are run it may be possible that worms using more than 34 rules may be identified, but this appears unlikely, because a large number of random worms has already been generated and rules are usually set early on. However some worms, such as worm 12053015626006367131237727 adds a new rule late on, as the 26th rule is set after 40,000 population.

### Recursive worm properties

**Figure 3:**
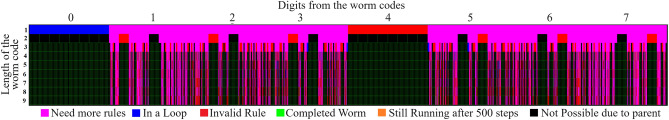
Diagram showing the categories of all possible worms up to a rule length of 9.

The diagram in Fig. 3 shows the category of all worm codes generated recursively to a rule length of 9. The diagram becomes difficult to interpret as the rule length increases, because the number of worms increases with the rule length. Therefore the rule length 9 worms are drawn at 175,000 worms per pixel so a lot of overlapping occurs on this diagram.

Using recursive search, all worms to a rule depth of 11 were simulated to population limit of 250,000 to identify their categories. This was a total of over 4.54 million worms (Table 1) and each worm was generated and saved as a .PNG image file. Over 97% of all worms are in category N. This is because most worms needed more than 11 rules before the population limit was reached. Other than category N, about 1.3% are in category T, 1.3% in category L, and 0.01% are in category R. The numbers at each rule length also include the sum of all parent branch worms. Simulating to a greater rule length than 11 in future would need additional computational power or hashlife algorithms.

It was found that the 1 worm in category R at rule length 7 (Table 1) is actually a looping shape builder, which is not automatically detected. This also applies to some worms in category R in the subsequent lengths from 8 to 11.

Using Eq. (1), the maximum number of unique worms was estimated as 70,311, up to and including rule length 7. In practice there were 28,305 worms (Table 1), which as expected does not exceeded the predicted maximum given by Eq. (1).

It was found that in recursive worms, more worms start with rule 1 (54%) than with rule 2 or 3 (22% each) (Table 2) in all worms up to rule length 10. With lower rule lengths, this affect is less, as with rule length 4, 36% start with rule 1. The proportion of worms starting with 1 may further increase above 54% as rule length increases beyond 10.

**Table 2:** More worms start with rule 1 than with rule 2 or 3.

	L8 (%)	L9 (%)	L10 (%)
1 as first rule	49.7	52.7	54.9
2 as first rule	24.2	22.9	22.1
3 as first rule	26.0	24.3	22.9

All 128 possible binary rules were observed being used, including all the 64 rules with an even number of paths available. Rules with an even number of available paths are rarer due to a maximum of 3 being used in a single worm. In recursive search, of all worms up to rule length of 10, all rules were activated. Some rules are used more often than others. The most common rule is 00001000, which is the first rule to activate and re-occurs whenever a worm is in an open space with all paths available. It makes up 12.7% of all rule activations in all worms up to rule length of 8.

### Random worm properties

**Figure 4:**
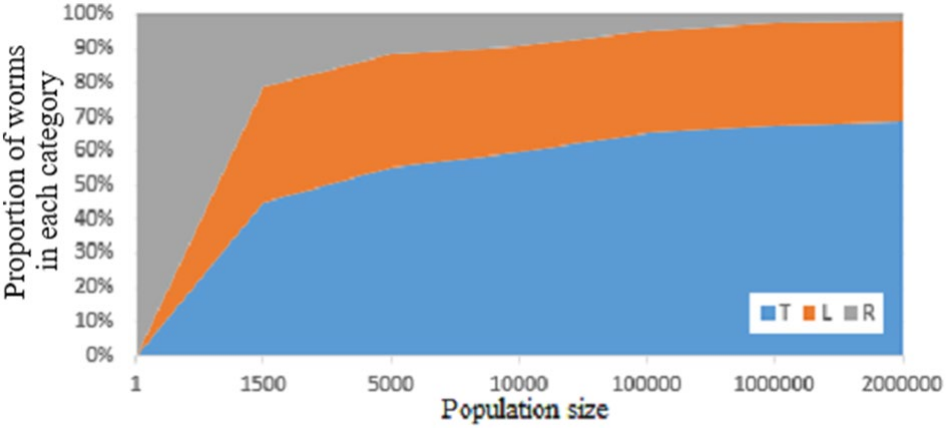
The proportion of random worms that end in each category by population size.

**Figure 5:**
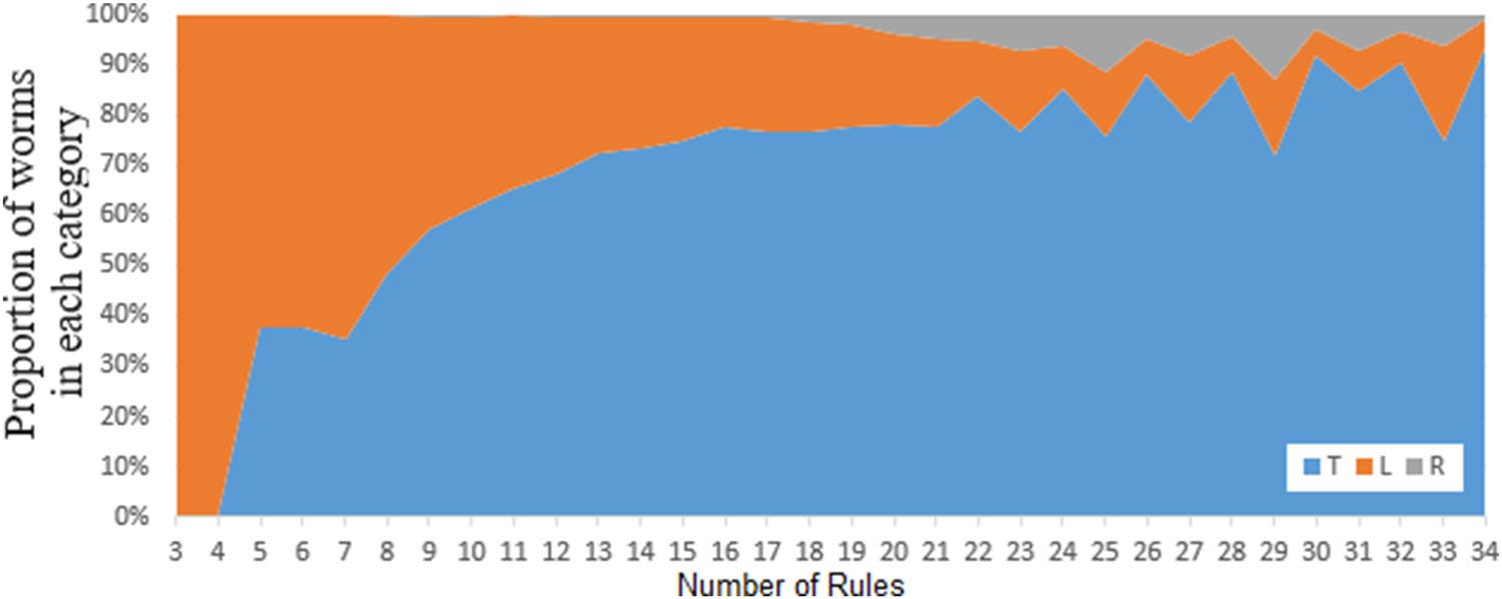
The proportion of random worms that end in each category by number of rules with 1 M population limit.

**Figure 6:**
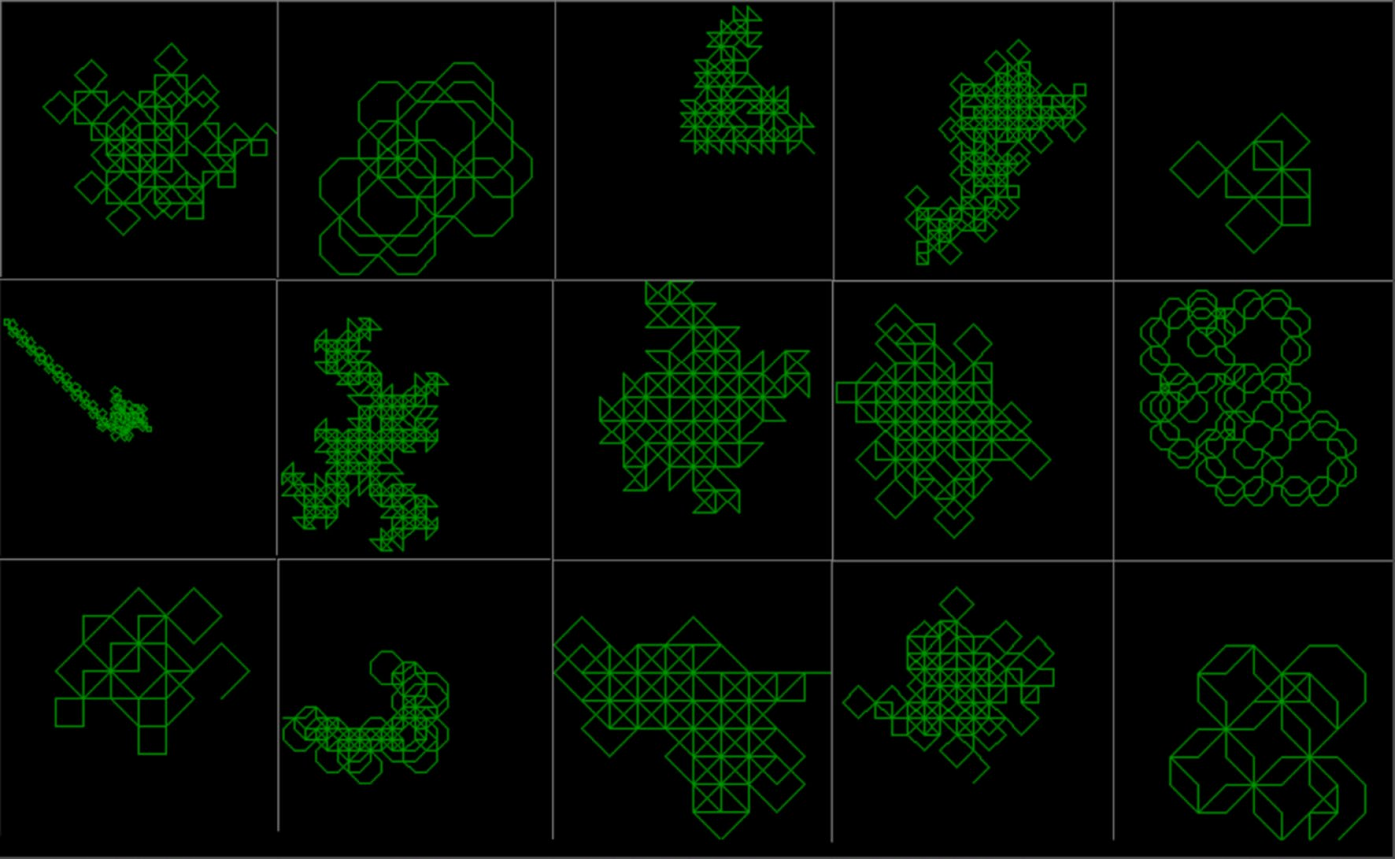
Fifteen worms found by random rule generation. Most are still running in category R with various population sizes below 2000.

Simulations were completed for 33,300 random worms such as the worms shown in Fig. 6 up to population limit of 2 million. The total proportion of worms that terminated T was 67%, the proportion of Looping L was 30% and still running R was less than 3%. Lower population limits were also evaluated in random worm searches to identify the proportion of worms that end with categories R, T or L. This is shown in Fig. 4 by population size up to 2 M. Figure 4 shows that as the population limit increases, the proportion of worms in category R decreases and the proportion of worms in category T increases, but the proportion of worms in category L stays roughly consistent. Figure 5 shows how the proportion in worm categories changes in worms of differing numbers of rules with 1 M population limit. Figure 5 shows that after running for 1 M steps, the worms that are most likely to still be category R have 25 or 29 rules. No worms with less than 16 rules were still running in category R at 1 M steps (Fig. 5). The zigzag pattern appears to be caused by differences in the proportions in each category between odd and even numbers of rules.

Properties of Category T random worms were analysed. Worms that terminate T are completed, so analysing their properties appears more meaningful. In comparison, category R worms have not yet completed so they may change category later on and category L worms are infinite and may have been stopped at some arbitrary limit.

In random worms terminating T under a 1 million population limit, population sizes ranged from 14–916,441 with mean of 12,761. Number of rules range from 5 to 34 (mean 21). This varies slightly from random worms including categories L and R with the same population limit (Table 1).

Random worms that terminate T are most likely to do so early on. For example, of all random worms that terminated T before 100,000, less than 1% did so in the between 50,000 and 100,000, such that 99% terminated T below 50,000. Similarly, in worms with 1 million population limit, only 2.5% had a population over 50,000 so 97.7% of worms had population in the lowest 5%. Also 99.5% terminated under 500,000, whereas only 0.5% of worms terminated T from 500,000 to 1 million.

In random worms that terminate T, the relationship of rule count to population count is not closely related. For example, some worms with 20 rules run beyond 10 million population, whereas some worms with 34 rules terminated T early with low population below 3000.

### Comparing characteristics of worms found by random and recursive searches

Worms are more likely to have an even number of rules than an odd number of rules. This applies to all worms selected randomly or recursively (Table 3). Population size also appeared to have slightly more chance of being even. The average population size of a worm was 37,926 for random worms, but in recursive worms the average population size depends on which starting worm is used.

**Table 3:** Comparison between characteristics of random and recursive worms, all to 1 M population limit.

	Recursive search (n = 9845) starting 1	Recursive search (n = 1331) starting from bimodal worm in Fig. 8b	Random search (n = 33,352)
Rule length—Min, max and average	1–24 (23.38)	18–26 (25.26)	3–34 (17.7)
Population size: min–max (average)	8–1,000,000 (4,499)	978–1,000,000 (70,035)	14–1,000,000 (37,926)
Proportion of worms with an odd number of rules	27.8%	35%	39%
Proportion of worms with an odd population size	49%	49%	30%

### Examples of worm images

Examples of fifteen worms found in random search are shown in Fig. 6. Most of these examples are still running in category R, with various small population sizes below 2000.

**Figure 7:**
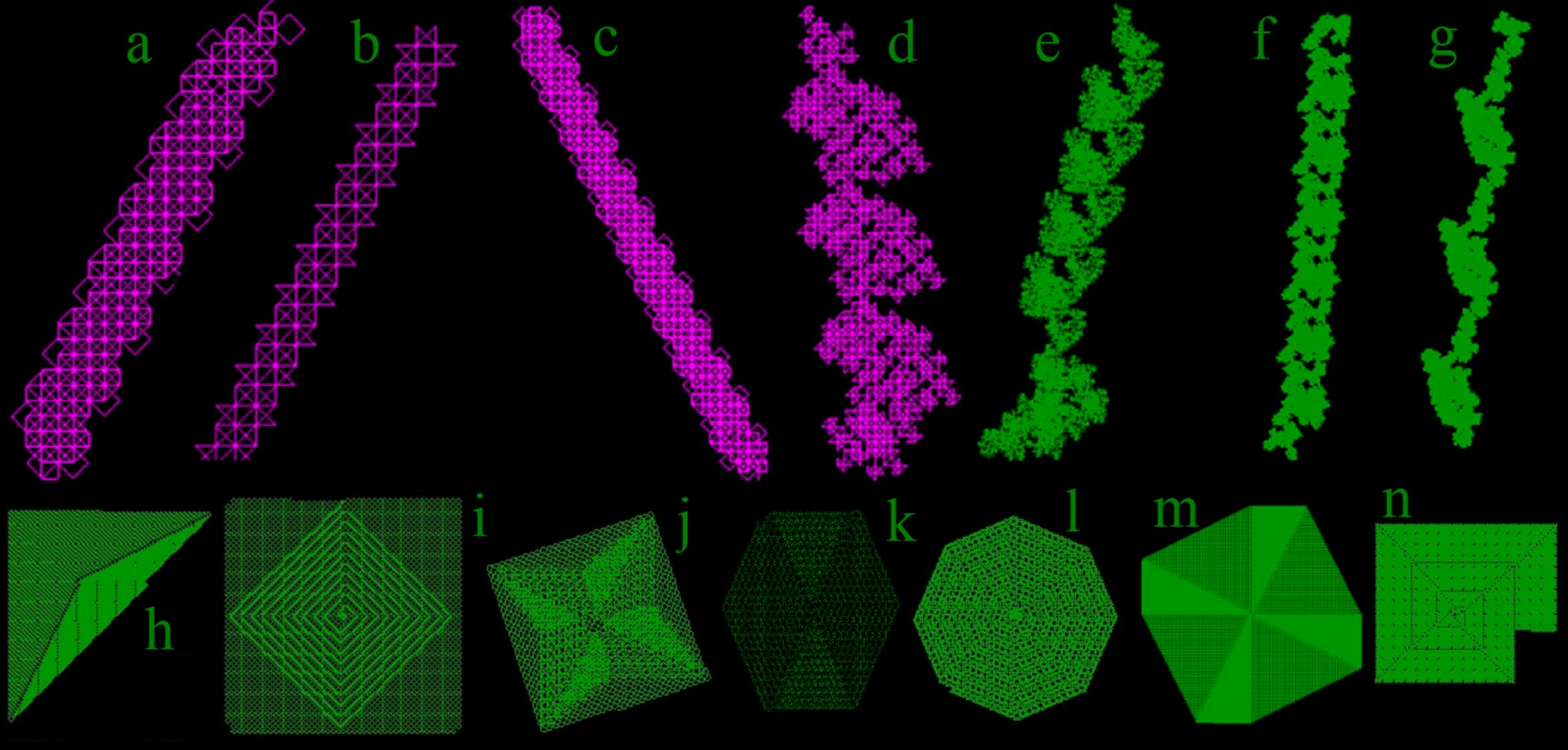
(**a**) ‘Shoot grower’ worm infinite loop (code 20502222252152221). (**b**) Pointy star grower with 9 rules rotated (code 552553555). (**c**) ‘Shoot grower’ worm with 17 rules (code 633306666636673767). (**d**) A loop of 484 (code 50275752501275776276353217). (**e**) A loop of size 10,310 with 24 rules (code 105372663603532736621176). (**f**) A loop of length 20,109 (code 1352527313371715661157). (**g**) A loop of size 42,621 (code 102727713362757175152736). (**h**) A triangle grower with 5 rules (code 16566). (**i**) Square grower with spiral inside (code 2176566567). (**j**) Square grower with star patterns (code 175316732673). (**k**) Hexagon grower (code 12622250737). (**l**) Octagon grower (code 1715166227). (**m**) Octagon spinner (code 331630707731625372). (**n**) spiral grower snail shell with 11 rules (code 31573150535).

Several shoot growers were identified (Fig. 7a–g) resembling those from triangular grids^4^. There are some even more basic loops with a length of only 4 lines in each repetitive segment.

**Figure 8:**
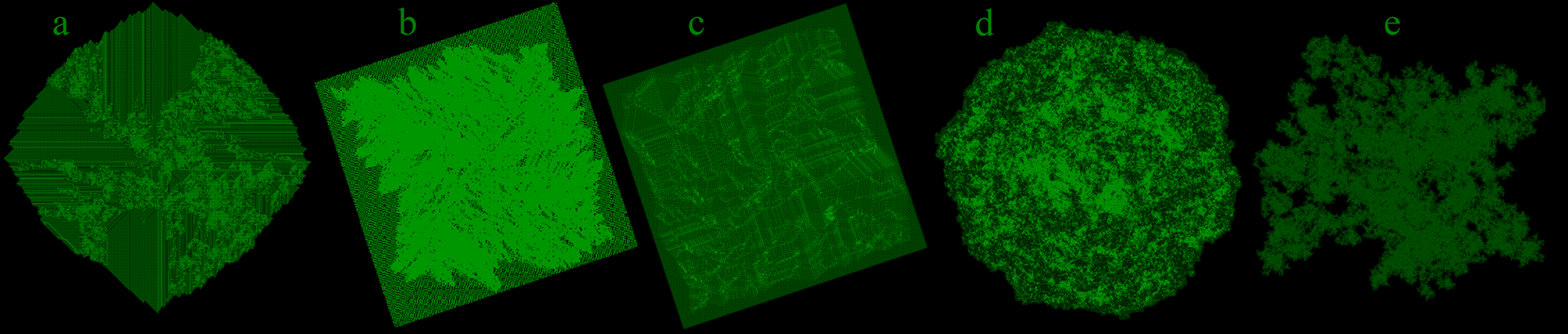
(**a**) Chaotic pattern with growing ordered sections at 1,000,000 steps (code 2531353756513537112062). (**b**,**c**) regular squares containing random patterns at 1,000,000 steps (codes 1571566002756310351671027 and 17500311512060116072057). (**d**) Growing unknown infinite path at 1,000,000 steps (code 15230365631501655775373337). (**e**) An irregular growing unknown/infinite path at 3,000,000 steps (code 270507163326720325110222771160).

Shape builders were found which produce triangles (Fig. 7h), squares (Fig. 7i,j), pentagons, hexagons (Fig. 7k), octagons (Fig. 7l,m), or other patterns such as stars (Fig. 7i) and spirals (Fig. 7n) and many other shapes, by adding consecutive layers to the outside at 45 or 22.5 degrees or intermediate angles. Several worms start by tracing chaotic patterns resembling circles (Fig. 8d). A few worms that look like Fig. 8d may suddenly enter a repetitive infinite loop at any time, which goes off to infinity in one direction, in worm 1302702170717267161075 this occurs after 306,000 population. Some loops are not entirely repetitive as the loop size grows over time with a repeating pattern, which consistently grows in size to infinity.

The most complex loop found so far has length of 42,621 (Fig. 7g). Longer loops still are sure to be found in longer runs.

Several worms were found to contain a mixture of order and chaos (Fig. 8a–c). Within this category, several particularly remarkable worms produced a regular square, containing irregular chaos (Fig. 8b,c). These worms oscillate irregularly between two behavioural modes, producing seemingly infinite patterns of ordered chaos. Over 1000 very similar variations of the worm in Fig. 8b were found by running a recursive search starting from the code of this worm.

Uncountable new worms found in this research still have an outcome which remains unknown (Fig. 8a–e). Many simulations continued running worms over 10 million population (Fig. 8e) with unpredictable chaotic movement, which may potentially continue infinitely or end at any moment. Many worms continue in category R beyond 10 million population. The majority of these grow in irregular shapes loosely resembling circles or clouds (Fig. 8d), but the paths of many other worms of unknown outcome also grow in very irregular shapes resembling extreme island coastlines (Fig. 8e). There remain substantial research tasks to explore larger proportions of the vast 10^33^ worm simulation genome, further categorising worm characteristics and path outcomes and employing further hashlife algorithms^9^ to speed up worm simulation.

### Interactive evolutionary computation

**Figure 9:**
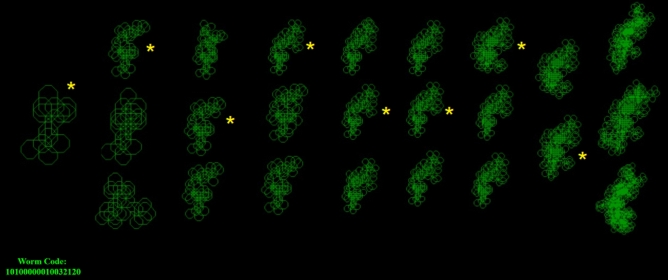
Worm Simulation code is available to run in web browser online^13^. This includes Interactive Evolutionary Computation (IEC). Parent worms are on the left and the asterisk indicates which parent the user has chosen at each step to produce the genetic code for subsequent generations, moving from left to right.

Several of the featured software implementations in java and C allow the user to interact with the rule setting process at runtime, to choose the worm’s next step. This is a form of Interactive Evolutionary Computation (IEC). In the web-based implementation (Fig. 9), the child worm can be selected by the user during runtime using IEC and is available to use through any web browser or device with no installation pre-requisites^13^. The provided code allows full reproduction of any worm code from this article and experimentation to identify new worm patterns. The result of IEC is that each child worm can be seen to build upon the shape of the previous generation’s path.

## Discussion

Many interesting worms remain in category R with unknown destiny that appear to continue chaotic movement potentially indefinitely. On the original Paterson’s worms hexagonal grid, only one worm remains in this category of unknown outcome (code 1042015)^8,9^. On this new proposed square grid, there are a huge number of worms of unknown outcome in category R. Of all the possible 10^33^ worms, it was found that approximately 2% continue running chaotically in category R beyond a million population size with unknown outcome, which currently leaves about 10^31^ worms with unknown outcome, for future research to explore.

To identify the final result of worms in category R and children of worms in category N, further investigation would be required with additional computing power or hashlife algorithms. The outcome of some worms in category T, L and I are known and completed, but there exist many more worms than could be examined.

When a number of worms are running, the timing with which they reach their final states appears to exhibit behaviour similar to a half-life, which can characterize rate of exponential decay. For example, all worms start in category R (Fig. 4). As the worms continue to run to larger population sizes, the number of worms remaining in category R decreases, or decays. The data in Fig. 4 appears to approximately suggest that each time the population limit is increased by an order of magnitude, the number of worms remaining in category R decays by half. It appears to be a possible outcome that all valid category R worms eventually decay to become either category L or T. A mathematical proof would be required to demonstrate this as it won’t be possible to simulate all 10^33^ worms, or to keep category R worms running for long enough.

The smallest worms to terminate complete in category T have a minimum population size of 14 and 5 rules. The smallest worms which enter infinite loops have 3 rules (Table 4).

**Table 4:** Minimum number of rules and size of worms in each category.

	T	L	R	I	N
Minimum population size	14	Infinite	1 (If population limit set to 1)	1	1
Minimum number of rules	5	3	1	1	0
Worm code	26326 (Fig. 2f)	337 and 377	1	4	None

Using the developed JavaScript website version, a random worm can easily be generated (Fig. 6) by pressing the key ‘g’. When a random worm is generated, it will be unique and will probably never be seen again. To find the exact same worm again would be equivalent to searching through every grain of sand on one hundred trillion earths. For example, none of the codes for the 15 worms in Fig. 1b or Fig. 6 were recorded, so it won’t be possible to find these exact worms again. This difficultly of recovering a worm’s genotype when given it’s phenotype increases further as rule length increases.

Worms have an even number of rules approximately twice as often as odd (Table 3) and this applies to any worms selected randomly or recursively. Random worms with over 20 rules are more likely to stay in category R for longer if they have an odd number of rules, indicated by the zigzag pattern in Fig. 5.

The majority (over 54%) of all worms start with rule 1 (Table 2), that is more common than worms starting with 2 and 3 combined. This proportion of worms starting with 1 increases beyond 55% as rule length increases beyond 10.

All 2^7^ = 128 of the possible binary rules from the worm’s 7 sensors were observed being used in worms of rule length 10 or less.

An Eq. (1) was proposed for estimating the maximum number of possible unique worms for any given rule length.

In random worms, the proportion of worms in each category is T: 67%, L: 30% and R: 3%, in 33,300 worms at population limit of 2 million (Fig. 4).

The proposed grid produces much more variety in the digital species of worm that can now be modelled, which haven’t been possible before. The previous worm system^5^ could model only 412 worms, whereas the proposed novel simulation environment with 8 path grid, can simulate a range of up to 10^33^ digital species of worm. This estimate may need further adjustments to increase accuracy due to other complex factors difficult to integrate into this estimation. To put into context, the total number of worms in this model is 10^33^ which is 100 trillion times more than the number of grains of sand on earth, which is roughly 10^18^. The number of worms is clearly very large since it approaches the number of atoms in the universe which is 10^78^. If there are 10^33^ worms, and if they could all be run at a speed of 10 worms per second, it would still take 3.17 * 10^23^ years to complete, which is twenty trillion times longer than the current age of the universe.

## Conclusion

These simulations and developed tools provide one of the clearest ways to visualise and quantify how local movement decisions lead to emergence of complex structured global search patterns. This contributes to explaining variations seen in fossil records (Fig. 1a) between the global search strategies of different species. This research also develops methods for categorising the emergent search behaviours into distinct categories by quantifying their properties.

This research offers the first published contributions to further develop Paterson’s worms since it was introduced over 50 years ago^5^.

In this model, decision rules are not set by the author, instead all possible sets of rules (10^33^ variations) within this particular environment emerge from a given environment definition. The purpose of visually and quantitatively analysing the outcomes of emergent worm patterns is that it enables conclusions to be made about how variation in resultant search strategies between species of virtual and real organisms relates to local movement decisions.

One of the interesting aspects among these findings is that some newly identified worms can switch between ordered and chaotic modes of movement. Future research could explore this further and aim to identify explanations, for how these simple local rules can lead to complex paths and behaviours including switching between ordered and chaotic movement patterns (Fig. 8b). This bi-modal, oscillating behaviour has not been identified before because it does not occur in any of the original Paterson’s worms models^7^. Within paths traced by these bimodal worms there are some further questions that could be investigated: what proportion of time is spent in the two modes? Are there other related yet undiscovered bimodal worms which spend more or less time in each mode? Are there any patterns within the durations spent in each mode?

These investigations and findings offer contributions to the field of Artificial life, studying biology by aiming to recreate aspects of biological phenomena, through the use of simulations with computer models. A conclusion that may be extrapolated to other creatures in addition to worms is that local movement decisions of a species appear to have substantial influence on determining the overall global search strategy that emerges.

## Data Availability

All of the worm codes for the worms shown in the figures of this paper are included within this paper. A collection of worm images and log files has been exported and is available from the author. This includes a set of worm path images for all worms up to a code length of 11. Log files from the random and recursive runs are available, enabling analysis of the categories of worm in both scenarios.

## Code availability

Source code in JavaScript for simulating worms is available and has been stored in an online source code repository^14^ with unique DOI under CC-BY licence. Readers can see this code running on the author’s website^13^ by using any compatible web browser on Windows, Apple or Unix, including Internet Explorer, Edge, Safari, Firefox, Google Chrome, including web browsers on Android or iOS smartphones.
